# DeepParcellation: A novel deep learning method for robust brain magnetic resonance imaging parcellation in older East Asians

**DOI:** 10.3389/fnagi.2022.1027857

**Published:** 2022-12-09

**Authors:** Eun-Cheon Lim, Uk-Su Choi, Kyu Yeong Choi, Jang Jae Lee, Yul-Wan Sung, Seiji Ogawa, Byeong Chae Kim, Kun Ho Lee, Jungsoo Gim, for The Alzheimer’s Disease Neuroimaging Initiative

**Affiliations:** ^1^Gwangju Alzheimer’s and Related Dementia Cohort Research Center, Chosun University, Gwangju, South Korea; ^2^BK FOUR Department of Integrative Biological Sciences, Chosun University, Gwangju, South Korea; ^3^Neurozen Inc., Seoul, South Korea; ^4^Medical Device Development Center, Daegu-Gyeongbuk Medical Innovation Foundation, Daegu, South Korea; ^5^Kansei Fukushi Research Institute, Tohoku Fukushi University, Sendai, Miyagi, Japan; ^6^Department of Neurology, Chonnam National University Medical School, Gwangju, South Korea; ^7^Department of Biomedical Science, Chosun University, Gwangju, South Korea; ^8^Korea Brain Research Institute, Daegu, South Korea

**Keywords:** deep learning, brain, 3D MRI, parcellation, DeepParcellation

## Abstract

Accurate parcellation of cortical regions is crucial for distinguishing morphometric changes in aged brains, particularly in degenerative brain diseases. Normal aging and neurodegeneration precipitate brain structural changes, leading to distinct tissue contrast and shape in people aged >60 years. Manual parcellation by trained radiologists can yield a highly accurate outline of the brain; however, analyzing large datasets is laborious and expensive. Alternatively, newly-developed computational models can quickly and accurately conduct brain parcellation, although thus far only for the brains of Caucasian individuals. To develop a computational model for the brain parcellation of older East Asians, we trained magnetic resonance images of dimensions 256 × 256 × 256 on 5,035 brains of older East Asians (Gwangju Alzheimer’s and Related Dementia) and 2,535 brains of Caucasians. The novel N-way strategy combining three memory reduction techniques inception blocks, dilated convolutions, and attention gates was adopted for our model to overcome the intrinsic memory requirement problem. Our method proved to be compatible with the commonly used parcellation model for Caucasians and showed higher similarity and robust reliability in older aged and East Asian groups. In addition, several brain regions showing the superiority of the parcellation suggest that DeepParcellation has a great potential for applications in neurodegenerative diseases such as Alzheimer’s disease.

**Figure:**
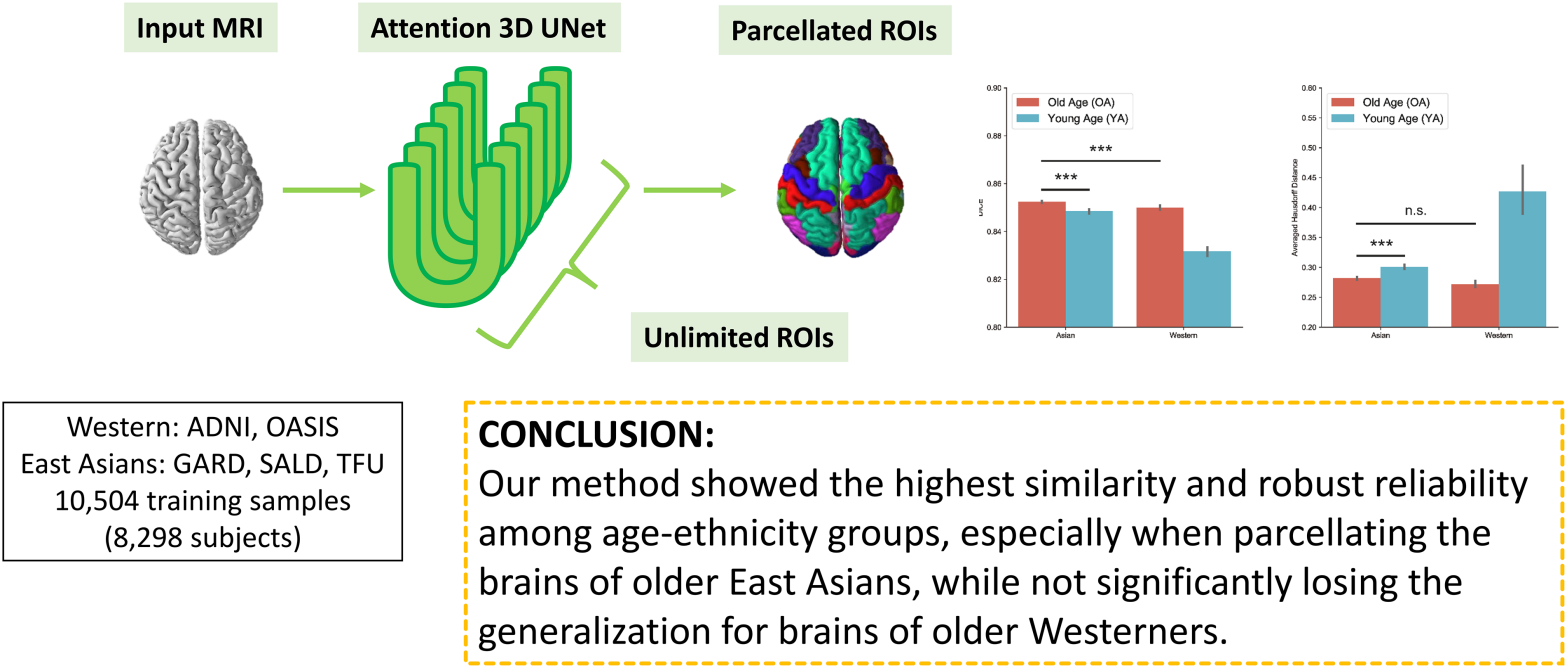
Graphical Abstract

## Introduction

Population growth, in association with aging, is a driving force for the increasing incidence of neurodegenerative diseases. Brain aging is reflected in structural changes and functional decline of the brain (Franke and Gaser, 2019). Estimating the brain’s biological age and monitoring the progression of age-related diseases (Wirth et al., 2013) demand accurate brain parcellation methods. However, earlier parcellation methods have overlooked aging morphology and ethnic differences, raising several concerns.

The first concern is that the brains of people aged >60 years show robustly different region-specific patterns compared with those of younger individuals (20–40s). For instance, the contrast between gray matter (GM) and white matter (WM) is usually higher in younger brains than in older brains because of changes in the amount of water in GM and WM tissues driven by myelin structural changes (Magnaldi et al., 1993). Subcortical structures show heterogeneous T1 and T2* values across regions due to changes in the composition of myelin and iron (Keuken et al., 2017). Abnormal volume and shape changes in the brain of older persons are observed as ventricular enlargement (Yue et al., 1997), WM hyperintensities (Habes et al., 2016), WM/GM atrophy (Oh et al., 2014), and heterogeneous subcortical brain volumes (Keuken et al., 2017).

The second concern is that brain volume and shape differ between East Asians and Caucasians (Chee et al., 2011). Brains οf Japanese individuals, for example, show morphological differences in the inferior parietal lobes, occipital regions, and posterior temporal regions compared with those of Europeans. The overall notion is that the brains of Japanese participants are shorter and wider than those of European participants (Zilles et al., 2001). A recent study validated the interethnic differences in cortical volume, cortical thickness, cortical surface area, and GM intensities (Tang et al., 2018), and reported that the brains of Chinese participants showed larger structural aspects in the temporal lobe and cingulate gyrus, but smaller ones in the parietal and frontal lobes than those in Caucasian individuals.

The final concern is related to computation time. There is a growing interest in collecting and studying brain MRI cohort data of East Asian individuals (Leong et al., 2017). A fast and reliable segmentation method is critical for such studies because conventional methods require long computation times to improve accuracy (Gronenschild et al., 2012). This can take many hours per brain, depending on computing power or algorithm complexity. Although recent advances have reduced the computation time, they are still not sufficient to handle big cohort data. To overcome these performance issues, deep learning approaches have recently been considered as a suitable solution in the neuroimaging field (Lee et al., 2011; Li et al., 2017; Roy et al., 2017; Rajchl et al., 2018; Thyreau et al., 2018; Thyreau and Taki, 2020). However, these models may not be directly applicable to brain parcellation of older East Asian individuals in terms of their runtime and accuracy, as they are based on brains of Caucasian individuals.

Deep learning models for brain segmentation and parcellation usually suffer from a tradeoff between the image dimensions and memory requirements. Based on image dimensions, models can be divided into four categories: 2D, 2.5D, partial 3D, and full 3D models. 2D models are the simplest, whereby only a single slice is segmented (Lee et al., 2011). They lose 3D contexts orthogonal to the selected plane and do not provide an aggregated 3D view of the parcellated regions of interest (ROIs). In contrast, 2.5D models attempt to reconstruct a 3D view from slice-wise segmentations (Henschel et al., 2020). This strategy could reduce some inconsistencies between slices by considering adjacent contexts. However, the aggregation could still create artifacts at random positions, degrading the overall accuracy (Henschel et al., 2020). Partial 3D models are the most common and use partial 3D images/patches derived from the whole image either by sub- or down-sampling. Partial 3D-based models can usually observe local 3D contexts, producing better parcellation for a certain area, while losing some global contexts (Li et al., 2017; Thyreau et al., 2018; Thyreau and Taki, 2020). A notable exception to this limitation is a cascaded model that can capture both global and local contexts by using downsampled and cropped images of the original resolution. However, this strategy is not capable of handling ROIs of varying sizes. Full 3D models can capture 3D contexts, intrinsically reducing inconsistency between slices and, in turn, potentially yielding high accuracy (Roy et al., 2017; Rajchl et al., 2018). However, the memory requirements become intractable, owing to the required increases in model parameters.

Region of interest number and size usually govern a model’s performance, mainly due to class imbalance. A model may predict a few ROIs of larger volumes with higher accuracy and shorter computing time than those segmenting several smaller ROIs. A few early models focused only on a single ROI, such as the hippocampus (Thyreau et al., 2018). Next-tier models can parcellate three representative tissues, including GM, WM, and cerebrospinal fluid (Chen et al., 2018), while finer-grained models collocate ROI predictions in the left and right cerebral hemispheres. Of particular interest is SkipDeconv-Net (SD-Net; Roy et al., 2017), which adopted UNet (Ronneberger et al., 2015) and DeconvNet (Roy et al., 2017). The SD-Net author introduced error corrective boosting (ECB), which updates high weights for classes with low accuracy per epoch, giving increased attention to those classes. However, ECB was applied only to the weighted cross-entropy and not to dice loss. A pioneering model handling >50 ROIs, which can segment MR slices into 56 classes using a 2D convolutional neural network (CNN), was introduced in 2011 (Lee et al., 2011). Some groups claimed to have successfully created a model that can parcellate >90 ROIs (Henschel et al., 2020; Thyreau and Taki, 2020). NeuroNet was trained on large-scale samples (*N* = 5,723) from the United Kingdom Biobank imaging study, and used three different segmentation tools, FSL, SPM, and MALP-EM, to generate label maps from T1-weighted images (Rajchl et al., 2018). However, there are some limitations to NeuroNet, such as the image dimensions of 128 × 128 × 128 and the difficulty to improve the low accuracy of some ROIs unless training is performed with weighted losses. Recently, FastSurferCNN achieved state-of-the-art performance in the parcellation of 95 ROIs using the 2.5D UNet with competitive dense blocks (Henschel et al., 2020). Notably, only a few models were trained and tested on >500 subjects in which the number of subjects is a very important factor in testing the reliability and validity of a given model (Rajchl et al., 2018; Henschel et al., 2020; Thyreau and Taki, 2020). A summary of the other available models is provided in Supplementary Tables A.1–A.4.

Accurate brain segmentation and parcellation are necessary for acquiring precise quantitative values of brain regions, including volume and cortical thickness (Ashburner and Friston, 2000; Chee et al., 2011; Tustison et al., 2014). These measurements have been used for brain age prediction (Franke and Gaser, 2019) and as biomarkers for neurodegenerative diseases including Parkinson’s disease (PD; Pagonabarraga et al., 2013) and Alzheimer’s disease (AD; Giorgio and Stefano, 2013).

In this study, we propose a novel 3D deep learning model, DeepParcellation, focusing on the brains of older East Asian individuals, which can parcellate 109 ROIs based on the Desikan-Killiany-Tourville (DKT) atlas. Our model employs 3D UNet architectures combined with inception blocks, dilated convolutions, and attention gates. The proposed model was robustly evaluated in (1) similarity of parcellated regions using dice coefficient (DICE), averaged Hausdorff Distance (aHD), (2) intra-subject reliability using the intra-class correlation coefficient (ICC), and (3) between-group variability between cognitively normal (CN) people and patients with AD.

## Materials and methods

All participants provided informed consent in accordance with the institutional review board of Chosun University Hospital, Republic of Korea.

### Experimental design

The primary aim of the study was to provide robust brain features for downstream analyses in studies of neurodegenerative diseases, aging, and biomarkers for monitoring patients in follow-ups. To enable support for unlimited number of ROIs, we introduced the N-way-weight strategy. Following the divide-and-conquer concept, we performed individual training for each ROI, avoiding competition during training so that ROI weights are independent. In addition, we integrated three memory reduction techniques to overcome a limitation in computational resources while retaining the full 3D characteristics: inception blocks, dilated convolutions, and attention gates.

To evaluate model performance, we collected brain MRI data of people of East Asian and Caucasian origins. We focused on similarity and robustness measures by which the accuracy and robustness of the downstream analyses could be improved.

### Model

#### Model background

UNet was initially developed for segmenting 2D biomedical images (Ronneberger et al., 2015). UNet follows an encoder–decoder structure for unsupervised learning, where the encoder (contracting path) captures global contexts, while the decoder (expanding path) performs detailed localizations. Skip connections in the expanding path combine contextual information and spatial locations.

A deep learning model can improve accuracy through a deeper or wider network structure. However, these structural changes lead to an increase in model capacity, causing overfitting and gradient vanishing problems. The Inception (or GoogLeNet) block mitigates these problems by introducing 1 × 1 or 1 × 1 × 1 convolution blocks, which reduce the number of feature maps while increasing depth (Szegedy et al., 2015).

In a convolutional layer, the kernel size determines the receptive field area, which represents the feature size. Multi-scale kernels can improve model performance, but also increase the number of parameters. Dilated convolution was introduced to enable larger receptive fields while maintaining the same number of parameters (Yu and Koltun, 2015). For instance, for a CNN with a kernel of size 3 × 3 and a dilation rate of 2, the receptive field becomes 5 × 5 while keeping the number of parameters to nine because every second row and column of the field will be skipped.

Attention is a mechanism that focuses more on features relevant to the target than on those less relevant (Bahdanau et al., 2015). Soft attention keeps attention on the global context, while hard attention observes a partial context, such as patches of an input. Soft attention can be implemented as a skip connection in UNet (Oktay et al., 2018).

#### Model details

**Figure 1:**
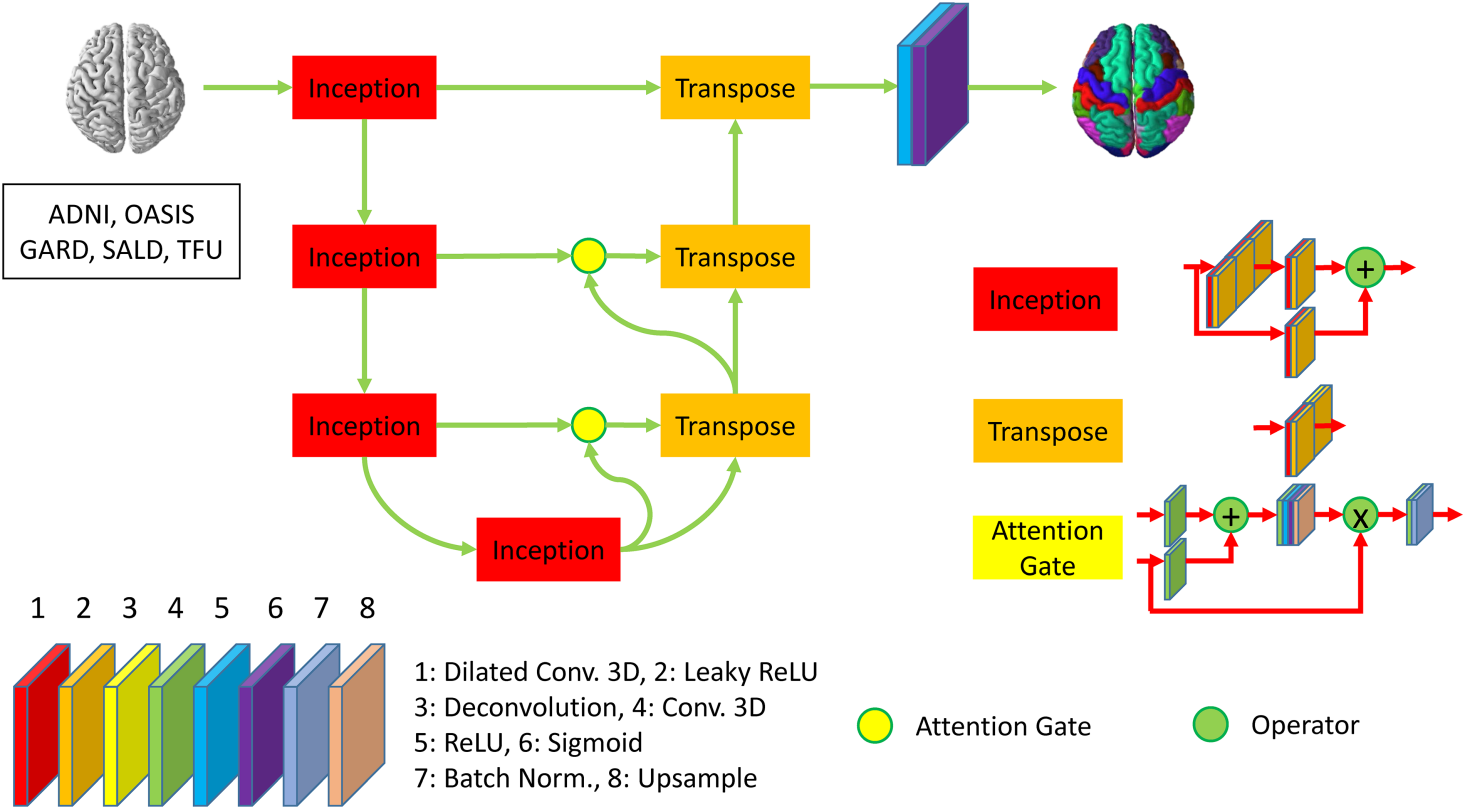
Description of DeepParcellation network architecture for a single region of interest. The network includes four inception blocks and three transpose blocks, which consist of eight different layers. The second, third, and fourth inception blocks are connected to transpose blocks through attention gates. The last transpose block is activated through ReLU and Sigmoid function to predict parcellated brain image.

We developed DeepParcellation using N-way multiple 3D UNet architectures combined with three memory reduction techniques inception blocks, dilated convolutions, and attention gates (Figure 1).

Our model was developed to overcome the intrinsic memory requirement problem of full 3D models by integrating parameter reduction techniques and splitting the weight (N-way weights). We adopted Inception blocks, dilated convolution, and attention gates, which reduced the number of model parameters and made an individual model trainable with limited memory capacity. N-way weights increase time linearly for training and predicting, but they enable the refinement of individual models through transfer learning, and further integrating heterogeneous models.

We have built a 3D UNet model using an input which is expected to be spatially matched to image resolutions of 256 × 256 × 256. The Encoder path is backed by four Inception blocks to reduce the number of feature maps, leading to the resolution of 32 × 32 × 32 at the bridge layer between the Encoder and the Decoder paths. The Decoder path is initiated by three 3D deconvolution (Transpose) blocks, where two initial Transpose blocks are connected with Attention gates. The Encoder and Decoder paths are mainly activated by the LeakyReLU. The output layer is encapsulated a 3D convolution layer with a sigmoid activation.

The model is packaged in the Python Package Index (PYPI) and can be deployed any Big data platforms or Cloud Computings, which support Python language, such as Amazon SageMaker, Google Cloud Vertex AI, and Microsoft Azure Machine Learning.

### Datasets

#### East Asian old age dataset

MR images of 5,035 older Koreans, aged >50 years, were collected from the Gwangju Alzheimer’s and Related Dementia (GARD) dataset (Table 1). GARD data were divided into 4,028, 503, and 504 subjects for training, validation, and test sets, respectively (Supplementary Table A.6). MRI data were acquired using 3.0 T (Skyra, Siemens, Munich, Germany) scanners. T1-MPRAGE sequences were acquired with the following parameters: repetition time (TR) = 2,300 ms, echo time (TE) = 2.14 ms, inversion time (TI) = 900 ms, field of view (FOV) = 256 × 256, and voxel size = 0.8 × 0.8 × 0.8 mm^3^. T2-SPACE sequence was acquired with the following parameters: TR = 2,300 ms, TE = 2.143 ms, TI = 900 ms, FOV = 256 × 256, and voxel size = 0.8 × 0.8 × 0.8 mm^3^.

**Table 1:** Characteristics of study samples.

**Dataset**	**No. of Subjects**	**Age**	**Male (%)**	**Female (%)**
GARD	5,035 (Sex unspecified: 51)	35–100 (72.62 ± 0.15)	40.62	58.37
SALD	487 (Sex unspecified: 2)	19–80 (45.31 ± 1.55)	36.76	62.83
TFU	140	18–22 (19.05 ± 0.14)	60	40
ADNI	956	55–97 (74.25 ± 0.46)	50.21	49.79
OASIS	1,579	18–98 (68.34 ± 0.68)	42.62	57.38

The distribution of age, sex, and sample size are described. ADNI, Alzheimer’s Disease Neuroimaging Initiative; GARD, Gwangju Alzheimer’s and Related Dementia; OASIS, Open Access Series of Imaging Studies; SALD, Southwest University Adult Lifespan Dataset; and TFU, Tohoku Fukushi University.

We used MR images of 116 Chinese individuals aged >60 years from the Southwest University Adult Lifespan Dataset (SALD) only for model evaluation. Details of the MRI protocol are described in the study by Wei et al. (2018).

#### East Asian young age dataset

MR images of 140 young Japanese individuals (mean age: 19.05, 18–22 years) were collected from the Tohoku Fukushi University (TFU) dataset (Sung et al., 2018). TFU MRI data were acquired using 3.0 T (Skyra, Siemens) scanners. T1-MPRAGE sequences were acquired with the following parameters: TR = 1,900 ms, TE = 2.52 ms, TI = 900 ms, FOV = 256 × 256, and voxel size = 1 × 1 × 1 mm^3^.

MR images of 154 young Chinese individuals <30 years from the SALD were used for model evaluation.

#### Caucasian OA dataset

MR images of 75 older Caucasians, aged >60 years, were collected from the Alzheimer’s Disease Neuroimaging Initiative (ADNI) dataset. Details of the dataset are described on the ADNI website.^1^

MR images of 149 older Caucasians, aged >60 years, were collected from the Open Access Series of Imaging Studies (OASIS) dataset. Details of the dataset are described on the OASIS website.^2^

#### Caucasian YA dataset

MR images of 107 young Caucasians <30 years were collected from the OASIS dataset. Details of the dataset are described on the OASIS website.^3^

#### Intra-subject reliability dataset

To evaluate intra-subject reliability among ethnicities, we used three young Japanese subjects with six repeated acquisitions (Sung et al., 2018) and three Caucasian subjects with 40-times repeated acquisitions within 31 days (Maclaren et al., 2014).

### Preparation for model training

The labeled images were reconstructed using T1 and T2 images to reduce topological mismatches when T2 images were available. To run the *recon-all* command with the T2 argument, T2-SPACE images were spatially registered to the T1 space, turning all different spatial resolutions to 256 × 256 × 256. We adopted the rigid-body registration strategy to minimize registration errors, because T1-MPRAGE and T2-SPACE were acquired from the same scanner. The registered T2-SPACE and T1-MPRAGE images were used as inputs for running the FreeSurfer *recon-all* procedure with the following commands:

*“recon-all -autorecon1 -i [path_to_input_T1] -T2 [path_to_aligned_T2] -T2pial -sd [output_dir] -s [subject_id].”*

*“recon-all -autorecon2 -T2 [path_to_aligned_T2] -sd [output_dir] -s [subject_id].”*

*“recon-all -autorecon3 -T2 [path_to_aligned_T2] -T2pial -sd [output_dir] -s [subject_id].”*

### Model training

We did not perform any data augmentation, which is very common for studies with a limited number of samples. The datasets were split into training, validation, and test sets (Supplementary Table A.6). We initially pre-trained 112 independent ROIs defined in automatic cortical parcellation and automatic segmentation volume with different numbers of rounds consisting of multiple epochs using Keras (Chollet, 2015) packages, mainly using six Tesla V-100 graphics processing units (GPUs) with 16 GB memory, and partially using six Tesla A-40 with 48 GB memory. Then, we performed transfer learning on 101 ROIs defined in the DKT atlas. Transfer learning is a learning strategy that reuses parts (or the whole) of the knowledge gained in previous tasks on a different but related task. Each epoch took approximately 1 h for 5,392 MRIs of a single ROI, and the model was trained for 121 days. The loss function improved curves near segmentation boundaries by using the DICE (Dice, 1945), voxel classification accuracy by assigning more weights to ROI masks according to class frequency between background and ROI voxels, and by using binary cross-entropy. We minimized the combined loss of binary cross-entropy and DICE using the Adam optimizer (Kingma and Ba, 2014). The learning rate was fixed to 0.0001, and the seed for the random number generator and optimizer weights were reset per round to overcome the local minima problem. Details of the training epoch information are shown in Supplementary Figure A.1.

### Aggregation of individual predictions

All N-ROI masks predicted by DeepParcellation require an aggregation step, which provides an integrated view of individual probability maps. A single predicted result represents a probability map of the ROI voxels after passing the input to the sigmoid function. Because all probability maps share identical dimensions, we can determine the most likely classes of every voxel in 3D coordinates. Given input ROIs, *x*, we calculate the final probability map using the softmax function (σ) as follows:


(1) $$\sigma \left(x_{j}\right)=\frac{e^{xj}}{\sum_{k}e^{x_{k}}}$$

where, input probability vector $x=\left\{x_{0},x_{1},\ldots ,x_{n-1}\right\}$ , and *k* is the number of ROIs.

### Statistical analysis

#### Similarity

Dice coefficient is a metric for evaluating segmentation accuracy. Given a binary mask of ground truth T and prediction P (voxels of the given class marked with 1 and background with 0), the DICE is defined as follows:


(2) $$DICE\left(T,P\right)=\frac{2T\cap P}{T+P}$$

The highest value for DICE is 1, which represents a situation when T and P are perfectly matched. DICE is a widely accepted metric because it allows direct observation of the similarity between T and P. However, DICE may not capture the variability in fundi of different sulci (or simply the curvature) around ROIs’ boundaries.

Yet another metric, the Hausdorff distance (HD), can be used to measure how far two surfaces are from each other, bridging the gap in DICE. Given ground truth G and segmentation S, the HD is defined as follows:


(3) $$HD\left(S,G\right)=\max\left(\begin{array}{c} \sup\\ s\in S \end{array}d\left(s,G\right),\begin{array}{c} \sup\\ g\in G \end{array}d\left(S,g\right)\right)$$

where sup represents the supremum or the greatest lower bound and is $d\left(x,Y\right)=\underset{y\in Y}{\inf}d\left(x,y\right)$ the distance x∈S from a point to the subset Y⊆S . Alternatively, the supremum distance or directed HD can be denoted as follows:


(4) $$h\left(A,B\right)=\begin{array}{c} maxmin\\ a\in A\;b\in B \end{array}∥a-b∥$$

where, norm (||) is the Euclidean distance. However, directed HD is prone to being affected by noise and outliers; therefore, we could take the aHD. To calculate aHD, we replace distance as follows:


(5) $$h\left(A,B\right)=\frac{1}{N}\underset{a\in A}{\sum }\min∥a-b∥$$

Equivalently, we can use a simplified equation of aHD as follows:


(6) $$aHD=\frac{\begin{array}{c} \sup\\ s\in S \end{array}d\left(s,G\right)+\begin{array}{c} \sup\\ g\in G \end{array}d\left(S,g\right)}{2}$$

We calculated DICE, and aHD for the different age groups (OA and YA) of East Asians and Caucasians by comparing the predicted masks with the outputs of FreeSurfer (ground truth).

To clearly observe metric differences between our proposed model and another method, FreeSurfer, we calculated fold changes using the mean measurements of the other method as the baseline, and Cohen’s d. Given two groups, Cohen’s d is calculated as follows:


(7) $$d=\frac{MEAN_{2}-MEAN_{1}}{SD_{pooled}}$$

(8) $$SD_{pooled}=\sqrt{\frac{STD_{1}^{2}-STD_{2}^{2}}{2}}$$

where, MEAN is the mean of a group and STD is a standard deviation of a group.

#### Intra-subject reliability

For test–retest reliability evaluation, we adopted ICC (2, *k*) with a two-way random-effects model (McGraw and Wong, 1996). The definition of ICC (2, *k*) is that randomly selected *k* raters rate each target, and the reliability is estimated for the average of *k* ratings. Thus, we defined repeated measurements of the number of voxels as raters and subjects as targets. We calculated ICCs using the test–retest dataset for brain volume measurement, with 18 MRI scans from three young Japanese subjects and 120 MRI scans from three young Caucasian subjects (Maclaren et al., 2014). ICCs were calculated using the voxel count of each ROI mask given by FreeSurfer and the proposed model.

#### Between-group variability evaluation

To evaluate the sensitivity to inter-group variations, we compared the normalized cortical volume of each ROI mask given by FreeSurfer and our model between East Asian CN and AD groups using the GARD dataset. The normalized volume was calculated by dividing the voxel number of individual ROIs by the total voxel counts of all parcellated regions. Independent *t*-tests and *f*-tests were conducted between groups.

## Results

### Similarity evaluation

**Figure 2:**
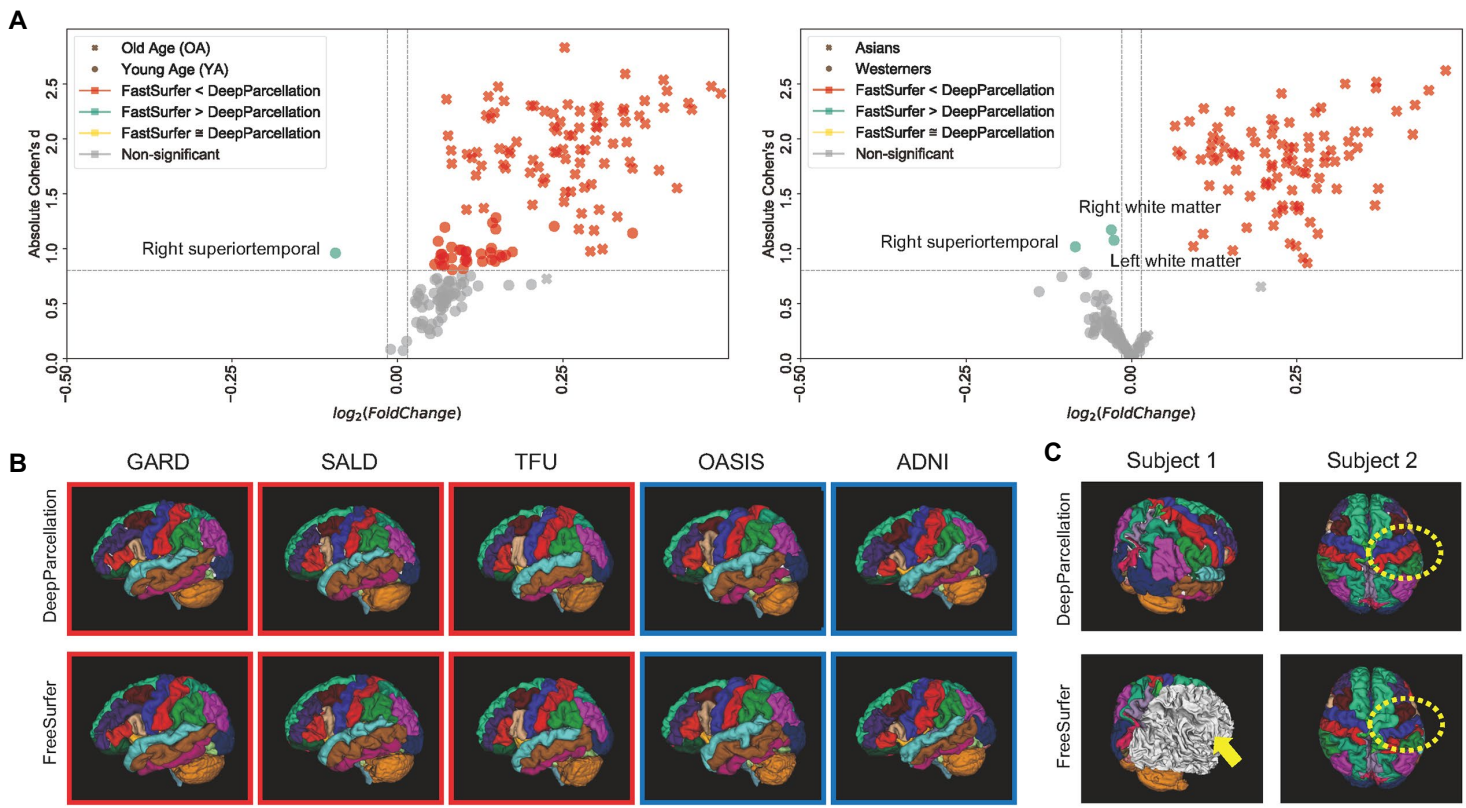
Performance of DeepParcellation. **(A)** Dice coefficient (DICE) comparison between DeepParcellation and FastSurfer. Using FreeSurfer output as surrogate for the ground truth, FastSurfer DICEs were baselines for calculating fold changes. Cohen’s *d* values were calculated using mean DICEs in two aspects (age and ethnicity). The horizontal line defines a Cohen’s *d* of 0.8, representing a large effect size. **(B)** Surface construction of parcellated brain images from representative subjects of different datasets. Red and blue squares indicate brains of Asians and Caucasians, respectively. **(C)** Examples of better parcellation with DeepParcellation compared with FreeSurfer. **(A)** Failures of right cortical parcellation in FreeSurfer (yellow arrows). **(B)** Wrong parcellation of right precentral and postcentral gyri in FreeSurfer (yellow dashed circle).

We calculated the DICEs by comparing the predicted ROIs of DeepParcellation and FastSurfer with the outputs of FreeSurfer version 7.1 as surrogates for the ground truth. FastSurfer was trained using FreeSurfer version 6.0, in a way that direct DICE comparisons between DeepParcellation and FastSurfer are infeasible. In this comparison, FreeSurfer version 7.1 outputs are likely to be unseen data from the FastSurfer model’s perspective; thus, the DICEs of FastSurfer serve as baselines for calculating fold changes. In the OA group, the mean DICEs for all 101 of DeepParcellation’s ROIs were higher than those of FastSurfer (Figure 2A). Similarly, higher mean DICE values were observed with DeepParcellation, except for two ROIs (left and right superior temporals) in the YA group. Specifically, FastSurfer showed a higher fold change than DeepParcellation only in the right superior temporal ROI with a large Cohen’s d value (Figure 2A). In the comparisons between ethnic groups, DeepParcellation showed higher similarities for all ROIs in the Asian group, while FastSurfer showed higher similarities for 74 out of 101 ROIs in the Caucasian group. With Cohen’s d criteria, DeepParcellation showed higher fold changes than FastSurfer except for three ROIs (left and right white matters and right superior temporal). We could not calculate the DICEs of six ROIs (CC_Posterior, CC_Anterior, CC_Mid_Posterior, CC_Mid_Anterior, CC_Central, and Optic-Chiasm) with FastSurfer because it did not produce ROI predictions with the default option.

**Figure 3:**
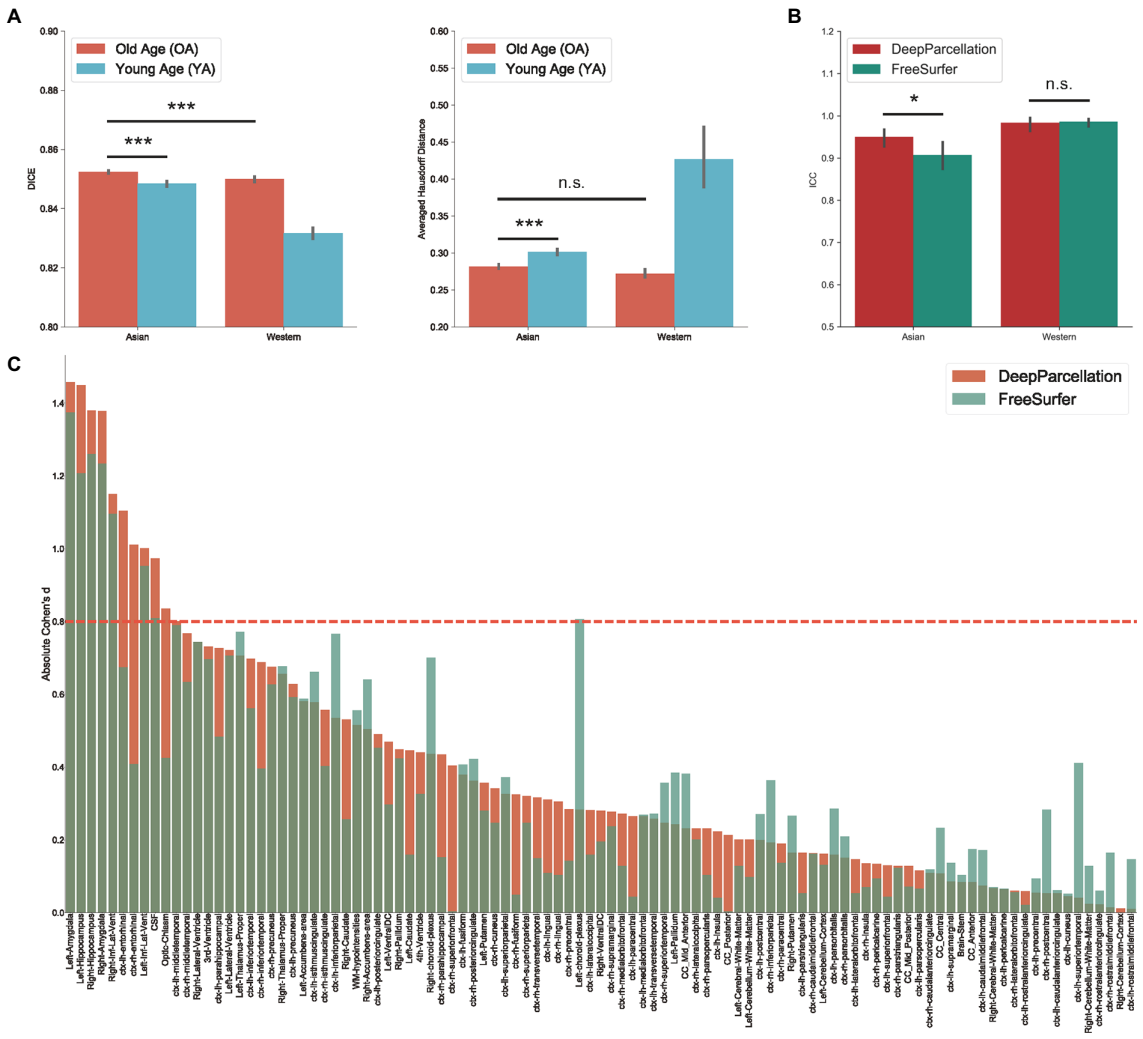
Evaluation of DeepParcellation. **(A)** Similarity evaluation among various age and ethnicity groups. Dice coefficient (DICE) and aHD comparison between groups. Brains of older Asian individuals showed significantly higher DICE values compared with brains of young Asian individuals and older Caucasian individuals. Brains of older Asian individuals also showed significantly higher averaged Hausdorff Distance values compared with brains of young Asian individuals. **(B)** Intra-subject reliability evaluation between DeepParcellation and FreeSurfer across ethnicities. DeepParcellation showed a significantly higher intra-class correlation coefficient than FreeSurfer in brains of Asians. **(C)** Between-group variability evaluation using Cohen’s ds between DeepParcellation and FreeSurfer across parcellated regions of interest. The red dashed line indicates a Cohen’s *d* of 0.8 (large effect size). n.s. not significant; * significant at *p* < 0.05; ** significant at *p* < 0.01; *** significant at *p* < 0.001.

We also compared the DICEs among different groups using only DeepParcellation predictions to observe the effects of age and ethnicity. The performance of DeepParcellation was superior on brains of East Asians to that in brains of Caucasian individuals, and on OA compared to YA datasets (Figure 3A). Among all groups, the overall highest average DICE (0.85) was observed in the East Asian OA group (one-way ANOVA and *post hoc* Tukey honestly significant difference, *p* < 0.001). In East Asians, the OA group showed a comparable number of brain regions with higher DICEs (43 out of 101 regions) to the YA group (Figure 3A). The aHD of the East Asian OA group (0.28) was significantly lower than that of the East Asian YA group (0.30; *post-hoc*, *p* < 0.001; Figure 3B) and Caucasian YA group (0.43; *post-hoc*, *p* < 0.001). In addition, the aHD of the East Asian YA group (0.30) was significantly lower than that of the Caucasian YA group (0.43; *post-hoc*, *p* < 0.001).

### Intra-subject reliability

DeepParcellation showed a significantly higher average ICC (0.95) than that of FreeSurfer (0.91) in East Asians (Figure 3B). There was no significant difference in DeepParcellation ICCs between East Asians and Caucasians (0.95 and 0.98, respectively), but significantly different FreeSurfer ICCs were observed between East Asians and Caucasians (0.91 and 0.99, respectively).

### Between-group variability

**Figure 4:**
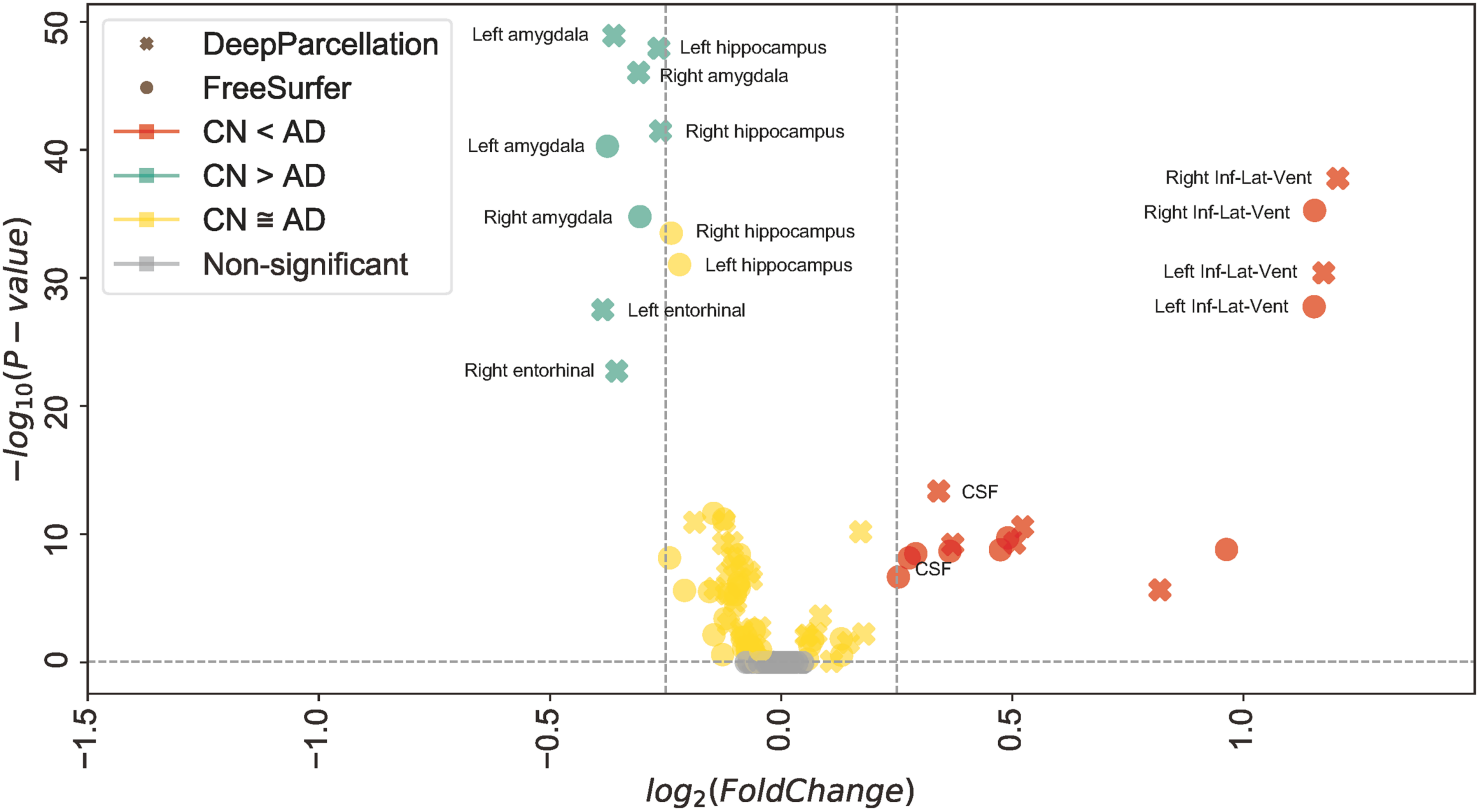
Robust dissociation of different diagnosed groups in DeepParcellation. Mean of normalized cortical volume with DeepParcellation (X) and FreeSurfer (O) in selected highly ranked regions sorted by the difference in the negative logarithm of *p* values between the cognitive normal (CN) and Alzheimer’s disease (AD) groups. DeepParcellation showed higher significance of normalized cortical volume differences than did FreeSurfer in regions highly associated with AD.

In 61 out of 101 regions, DeepParcellation achieved higher statistical power of group differences between the CN and AD groups compared to FreeSurfer (Figures 3C, 4). We selected nine highly ranked regions sorted by the difference in the negative logarithm of *p* values between CN and AD groups: bilateral entorhinal cortex, bilateral amygdala, bilateral hippocampus, and bilateral inferior lateral ventricles (Figure 4).

### Processing success rate of DeepParcellation and FreeSurfer

We reported the success rate of DeepParcellation and FreeSurfer with default *recon-all* commands across different datasets (Table 2).

**Table 2:** Processing success rate of DeepParcellation and Freesurfer showing absolute numbers and percentage.

**Dataset**	**Module**	**Count**	**Total**	**Success Rate (%)**
ADNI	DeepParcellation	4,500	4,502	99.96
ADNI	FreeSurfer	4,461	4,502	99.09
OASIS	DeepParcellation	5,162	5,162	100
OASIS	FreeSurfer	4,889	5,162	94.71
GARD	DeepParcellation	7,337	7,337	100
GARD	FreeSurfer	7,337	7,337	100
SALD	DeepParcellation	494	494	100
SALD	FreeSurfer	487	494	98.58
TFU	DeepParcellation	153	153	100
TFU	FreeSurfer	152	153	99.35

ADNI, Alzheimer’s Disease Neuroimaging Initiative; GARD, Gwangju Alzheimer’s & Related Dementia; OASIS, Open Access Series of Imaging Studies; SALD, Southwest University Adult Lifespan Dataset; TFU, Tohoku Fukushi University.

DeepParcellation failed only in two subjects in the ADNI dataset and succeeded in all other subjects in the other datasets. On the other hand, FreeSurfer failed in some subjects in all datasets (41, 273, 41, 7, and 1 subject in the ADNI, OASIS, GARD, SALD, and TFU datasets, respectively).

Most subjects were successfully processed by both DeepParcellation and FreeSurfer (Figure 2B; Supplementary Figure A.3A), but showed relatively lower average DICEs. We found that lower DICEs in FreeSurfer were due to failures in parcellations and misannotations of several brain regions (Figure 2C; Supplementary Figure A.3B).

### Runtime

We reported the runtime of DeepParcellation and FreeSurfer with the default *recon-all* command. DeepParcellation consistently performed full parcellation in about 2 min 30 s per sample, using a single GPU. The runtime of DeepParcellation using CPUs (central processing units) depends on the CPU number, but it did not improve when it was higher than the ROI number. The runtime of FreeSurfer fluctuated, with a median time of approximately 13 and 9 h using a single CPU and 24 CPUs, respectively (Supplementary Table A.5).

## Discussion

Herein, we proposed a novel full 3D deep learning model for automatic brain MRI parcellation that shows comparable or better performance in terms of similarity and reliability for the brain of older East Asian individuals than an existing model, FreeSurfer. Previous deep learning models have utilized several tens or hundreds of samples of brains belonging to Caucasian individuals (Rajchl et al., 2018; Thyreau and Taki, 2020), as East Asian cohort datasets were not sufficiently established or not publicly available, with the exception of a few cases (Chee et al., 2009; Wei et al., 2018). These East Asian studies consisted of hundreds of subjects, but the sample size may not be sufficient to train a deep learning model for older East Asian individuals. In contrast, the sample size (GARD, *N* = 7,166) and age range (mean age: 72.62, 35–100 years) of our study were suitable for implementing a deep learning model representing older East Asian individuals.

Our model was trained using the full 3D context. This forgoes the need for aggregating contexts of three orthogonal planes, meaning that the model may have a higher potential to achieve better accuracy. Our model was developed to overcome the intrinsic memory requirement problem of full 3D models by integrating parameter reduction techniques, such as inception blocks, dilated convolution, attention gates, and weight splitting (N-way weights). The N-way weight strategy increases the time needed for training and prediction, but enables individual model refinement through transfer learning, and allows integration of heterogeneous models. In contrast, 2.5D models require training and prediction of three models for each image plane. They can utilize a mini-batch strategy with a batch size larger than that of a full 3D model, thereby improving runtime. However, 2D or 2.5D models may lose 3D contexts, which influences parcellation accuracy.

With a classical model predicting multiple ROIs (e.g., through cross-entropy), re-weighting is rarely possible due to increased competition while minimizing the loss of multiple ROIs, where non-linear interventions occur in the model parameter space. Thus, in such cases, one should start the training from scratch, although convergence with the changed ROI configuration is not guaranteed. In contrast, competition during training never occurs with the N-way-weight strategy because weights are independent of each other.

The integration of models with heterogeneous structures is generally infeasible. We found that 3D UNet attains lower average DICEs for vessel parcellations, owing to geometrical uncertainty and randomness in shapes. With other network structures, such as partial 3D models, one may overcome uncertainty, but integration incurs re-designing and re-training of a classical model. In contrast, the N-way-weight strategy allows for individual model replacement; thus, it is possible to improve vessel parcellations without disturbing the probability map of other ROIs.

Most deep learning models for brain parcellation have been oriented toward Caucasian people (Thyreau and Taki, 2020). As there are several differences in anatomy between brains of East Asian and Caucasian individuals, including shape and volume, our model can yield a better prediction, especially in the brains of older East Asian individuals, for the following reasons:

First, DeepParcellation showed robustly higher similarities in the East Asian OA group than in the other groups. That the highest DICE and lowest aHD were observed in the East Asian OA group indicates that our model is optimized for older East Asians. In the DICE evaluation, age-related dominant structural changes were observed in East Asians, although a similar number of regions showed higher DICEs between the OA and YA groups (Supplementary Figure A.1). The OA group showed higher DICEs in age-related brain regions such as the ventricles (Yue et al., 1997), and WM hypointensities (Habes et al., 2016) compared to the YA group. Structural changes in these regions are closely associated with aging and neurodegenerative diseases, such as AD (Almkvist et al., 1992).

Second, DeepParcellation showed higher intra-subject reliability than FreeSurfer. The higher ICC of our model (0.95 vs. 0.91) indicates that DeepParcellation consistently parcellates East Asian brains. Since our model learned a global distribution of East Asian brain patterns such as shape, intensity, contrast, and volume from several thousands of East Asian samples, the predictions of unseen data are superior to those of FreeSurfer. The non-significant difference in ICCs between DeepParcellation and FreeSurfer in brains of Caucasians supports the good generalizability of the model.

Third, DeepParcellation showed better sensitivity to group differences in East Asians than did FreeSurfer. The higher statistical metrics displayed by our model were derived from significantly different mean values and variations of the normalized volume between cognitive groups (CN and AD). DeepParcellation was superior to FreeSurfer in terms of absolute Cohen’s d in selected regions (the bilateral entorhinal cortex, bilateral amygdala, bilateral hippocampus, and bilateral inferior lateral ventricles; Figure 3C). These regions showed significant differences in mean values and variations in comparison to FreeSurfer (Figure 4). The difference was more robustly observed in the CN group than in the AD group because our model training was performed on more East Asian CN samples. Of particular significance is the fact that the selected regions belong to the medial temporal lobe, which is highly associated with AD (Dickerson et al., 2001). Volume changes in these regions have been used to develop biomarkers for AD diagnosis and cognitive decline (Wirth et al., 2013).

Finally, DeepParcellation shows a high success rate for parcellation. Some MRI data can display an abnormal intensity distribution or shape beyond the normal range of their population. Using the default command (*recon-all*), FreeSurfer can fail to process such a brain image because of inhomogeneous intensity ranges or mismatched coordinates with respect to a standard template. Contrarily, our model learned geographic patterns and image properties of brain structures from thousands of samples, rather than performing a sequence of manual algorithms. DeepParcellation, therefore, increases the chances of making valid predictions for atypical brains where FreeSurfer could fail.

Our model has some limitations that should be overcome in further studies. First, we did not train the model with different MRI acquisition parameters from multiple vendors. Most data came from our in-house GARD dataset based on the same scanner and acquisition parameters; thus, our model may have lacked technical generalization. Although our model already showed good performance on brains of Caucasians from the ADNI and OASIS databases, we think that it would be better to create a specialized model for the brains of Caucasian individuals rather than pursuing generalization, considering the anatomical differences between brains of East Asians and Caucasians. Second, segmentation failures at ROI boundaries can more severely influence smaller ROIs, which is a class imbalance problem. We may reweigh the probability values by refining some problematic ROIs before passing them to the final softmax function. Alternatively, we could replace certain predictions with newer ones by introducing a heterogeneous network with higher accuracy than that of our base 3D Attention UNet model.

Despite these limitations, DeepParcellation has great potential for use in neuroimaging studies. The predicted parcellation derived from our method can be extended to neurodevelopmental and clinical studies, such as brain age prediction or biomarker development for neurodegenerative diseases. Parcellated subcortical regions, including the putamen, caudate, and hippocampus, predict brain age with good accuracy (Zhao et al., 2019). Decreased cortical thickness in temporal regions was found in patients with PD (Pagonabarraga et al., 2013) and reduced volumes in cortical regions were reported in patients with AD (Wirth et al., 2013). Since aging affects certain brain regions differently (Toga et al., 2006), accurate and precise structural measurements are critical for monitoring neurodegenerative processes. Our robust and reliable parcellation method of the brain of older individuals can guarantee higher prediction accuracy and help disease diagnosis.

The fast and robust parcellation achieved by our proposed model can accelerate big brain MRI data analysis. Our method provides crucial data for secondary applications, such as early detection or monitoring the progress of neurodegenerative diseases.

## Data availability statement

The raw data supporting the conclusions of this article will be made available by the authors, without undue reservation.

## Ethics statement

The studies involving human participants were reviewed and approved by Institutional review board of Chosun University Hospital, Republic of Korea. The patients/participants provided their written informed consent to participate in this study.

## Author contributions

E-CL and U-SC: conceptualization, formal analysis, investigation, visualization, and writing—original draft. E-CL: data curation, methodology, and software. KL: funding acquisition. E-CL, U-SC, and JG: project administration. KL, KC, JL, BK, Y-WS, SO, and for The Alzheimer’s Disease Neuroimaging Initiative: resources. JG, KL, Y-WS, and SO: supervision. E-CL, U-SC, JG, and KL: writing—review and editing. All authors contributed to the article and approved the submitted version.

## Funding

This research was supported by the Original Technology Research Program for Brain Science of the National Research Foundation (NRF) was funded by the Korean government, MSIT grant NRF-2014M3C7A1046041 (KL); the Healthcare AI Convergence Research & Development Program through the National IT Industry Promotion Agency of Korea (No.1711120216) and the KBRI basic research program through Korea Brain Research Institute funded by the Ministry of Science and ICT grant 21-BR-03-05 (KL); the National Institute on Aging of the National Institutes of Health under Award Number U01AG062602 (KL). Data collection and sharing for this project was partially funded by the ADNI (National Institutes of Health Grant U01 AG024904) and DOD ADNI (Department of Defense award number W81XWH-12-2- 0012). ADNI is funded by the National Institute on Aging, the National Institute of Biomedical Imaging and Bioengineering, and through generous contributions from the following: AbbVie, Alzheimer’s Association; Alzheimer’s Drug Discovery Foundation; Araclon Biotech; BioClinica, Inc.; Biogen; Bristol-Myers Squibb Company; CereSpir, Inc.; Cogstate; Eisai Inc.; Elan Pharmaceuticals, Inc.; Eli Lilly and Company; EuroImmun; F. Hoffmann-La Roche Ltd. and its affiliated company Genentech, Inc.; Fujirebio; GE Healthcare; IXICO Ltd.; Janssen Alzheimer Immunotherapy Research & Development, LLC.; Johnson & Johnson Pharmaceutical Research & Development, LLC.; Lumosity; Lundbeck; Merck & Co., Inc.; Meso Scale Diagnostics, LLC.; NeuroRx Research; Neurotrack Technologies; Novartis Pharmaceuticals Corporation; Pfizer Inc.; Piramal Imaging; Servier; Takeda Pharmaceutical Company; and Transition Therapeutics. The Canadian Institutes of Health Research are providing funds to support ADNI clinical sites in Canada. Private sector contributions are facilitated by the Foundation for the National Institutes of Health (www.fnih.org). The grantee organization is the Northern California Institute for Research and Education, and the study is coordinated by the Alzheimer’s Therapeutic Research Institute at the University of Southern California. ADNI data are disseminated by the Laboratory for Neuro Imaging at the University of Southern California.

## Conflict of interest

KL was employed by Neurozen Inc.

The remaining authors declare that the research was conducted in the absence of any commercial or financial relationships that could be construed as a potential conflict of interest.

## Publisher’s note

All claims expressed in this article are solely those of the authors and do not necessarily represent those of their affiliated organizations, or those of the publisher, the editors and the reviewers. Any product that may be evaluated in this article, or claim that may be made by its manufacturer, is not guaranteed or endorsed by the publisher.

## Footnotes

^1^ http://adni.loni.usc.edu

^2^ https://www.oasis-brains.org

^3^ https://www.oasis-brains.org

## Abbreviations

AD, Alzheimer’s disease
ADNI, Alzheimer’s disease neuroimaging initiative
aHD, Averaged Hausdorff Distance
CN, Cognitively normal
CNN, Convolutional neural network
CPU, Central processing unit
DICE, Dice coefficient
DKT, Desikan-Killiany-Tourville
ECB, error corrective boosting
FOV, Field of view
GARD, Gwangju Alzheimer’s and related dementia
GM, Gray matter
GPU, Graphics processing unit
HD, Hausdorff distance
ICC, Intra-class correlation coefficient
MRI, magnetic resonance imaging
OA, old age
OASIS, Open access series of imaging studies
PD, Parkinson’s disease
ROI, region of interest
SALD, Southwest university adult lifespan dataset
TE, Time of echo
TFU, Tohoku Fukushi University
TI, Time of inversion
TR, Time of repetition
WM, White matter
YA, Young age
