# Supplementary material for: DeepParcellation: A novel deep learning method for robust brain magnetic resonance imaging parcellation in older East Asians
Source: Front Aging Neurosci. 2022 Dec 9;14:1027857. doi: 10.3389/fnagi.2022.1027857 (PMC9783623; doi:10.3389/fnagi.2022.1027857)
Supplement: Supplementary file 1 [file Data_Sheet_1.pdf]

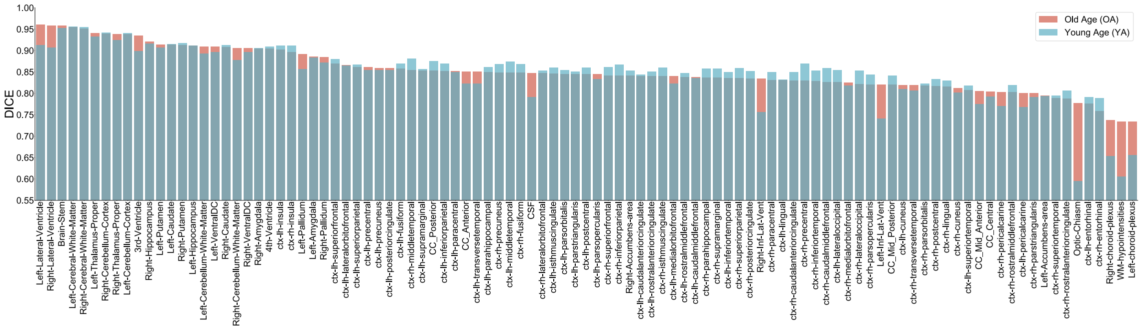

Figure A.1 DICEs of each ROI in the age group

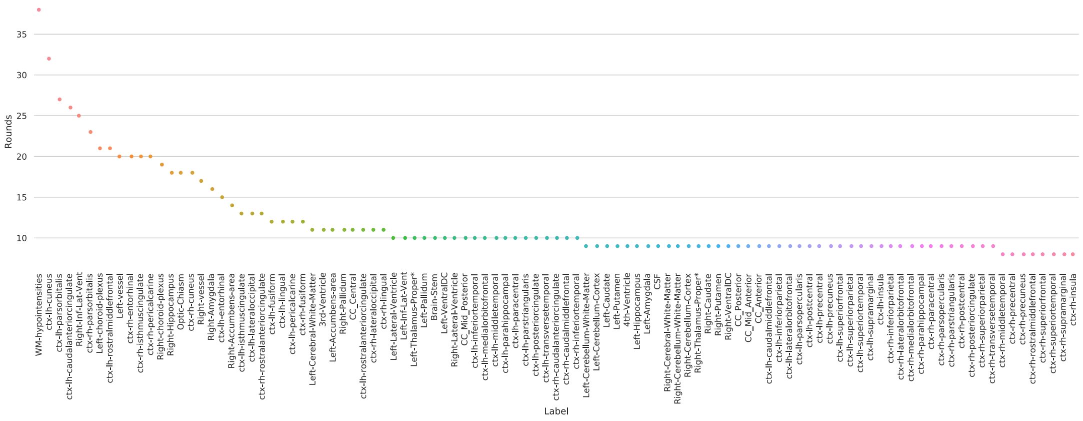

Figure A.2 The number of training epochs. Each ROI requires the different number of epochs in order to reach at a loss convergence.

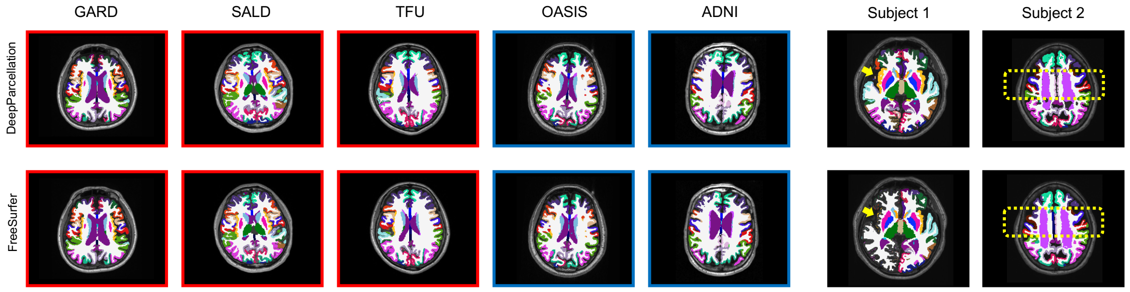

Figure A.3 Volumetric construction of parcellated brain images from the representative subjects of different datasets (first five columns). Red and blue squares indicate Asian brains and Western brains, respectively. Examples of better parcellation of DeepParcellation compared with FreeSurfer (last two columns). (A) Failures of the right cortical parcellation in FreeSurfer (yellow arrows). (B) Wrong parcellation of right precentral and postcentral gyri in FreeSurfer (yellow dashed rectangles).

Table A.1. Summary of deep learning models for brain MRI segmentation of a single ROI

Name	Backend	Image Resolution	# ROIs	N (train/validation/test)	Note	Reference
	3D FCNN	160x256x256	1	15/NA/5	Down-sampled image	[1]
CompNet	2.5D custom Encoder-Decoder	256x256	1	203/NA/203	Tumor lesion	[2]
Hippodeep	3D FCNN	48x72x64	1	2500/NA/659	Cropped image Preprocessing required	[3]

Table A.2. Summary of deep learning models for brain MRI segmentation of three important tissues (WM, GM, and CSF)

Name	Backend	Image Resolution	# ROIs	N (train/validation/test)	Note	Reference
	2D CNN	256x256	3	7/NA/1	Infant brain	[4]
Pyramid-LSTM	LSTM	240x240x25 cubes	3	5/NA/15	3 modalities Extra preprocessing	[5]
VoxResNet	3D CNN	80x80x48 cubes 3 modalities	4	25/NA/10	Residual function Multi-modality	[6]
MMAN	2.5D CNN	240x240 3 modalities	3	4/1/15	Dilation Inception	[7]

Table A.3. Summary of deep learning models for brain MRI segmentation of 8-30 ROIs

Name	Backend	Image Resolution	# ROIs	N (train/validation/test)	Note	Reference
Brainseg	2D CNN	256x256	8	15/NA/14	Only works at axial axis	[8]
Hough-CNN	2D,2.5D,3D CNN	31<br>31x31<br>31x31x31 cubes	26	8/NA/24	3D model shows better accuracy	[9]
Mesh-Net	3D FCNN	256x256x256	3, 8	20/2/100<br>5/NA/15<br>3/1/1	Dilation<br>Unexpected artifacts	[10]
SD-Net	3D UNet	256x256x64	26, 24	581+15/5/10<br>581+10/5/5	Deconvolution<br>FCNN<br>Error corrective boosting	[11]
DeepNAT	3D CNN	23x23x23 cubes	25	20/NA/10	Multi-task learning, hierarchical segmentation, spectral coordinates,	[12]

					conditional random field	
3D FCNN BrainStruct	3D FCNN	256x256x256	8	150/NA/923	Extra pre-/post-processing	[13]
QuickNAT	2.5D CNN	256x256	27	581+28/NA/191	Aggregation Median frequency balancing	[14]
PSACNN	3D UNet+CNN	96x96x96 cubes	3,9,12	16/3/94	Synthesize multi-modality images	[15]
SW-3D-UNet	3D UNet	32x32x32 cubes	3, 25	4/1/15<br>20/NA/10	Losing some 3D contexts	[16]

Table A.4. Summary of deep learning models for brain MRI segmentation of over 50 ROIs

Name	Backend	Image Resolution	# ROIs	N (train/validation/test)	Note	Reference
	2D CNN	28x28 + 14x14x4 patches	56	20/1/19		[17]
SegNet	2D+3D CNN	29x29x3 patches + 13x13x13 cubes	134	30/NA/5 (approx..)		[18]
	2D CNN	25x25,51x51,75x75 patches	134	10/NA/20	Infant Losing global context	[19]

HighRes3DNet	3D CNN	96x96x96 cubes	155	443/50/50	Dilation Residual Function	[20]
BrainSegNet	2D+3D CNN	31x31x6+21x21x21x2 cubes	134<br>32<br>54<br>67<br>83	15/NA/20<br>9/NA/9<br>20/NA/20<br>10/NA/10<br>15/NA/15	Multi-filter CNN	[21]
NeuroNet	3D Encoder-Decoder	128x128x128	139	5000/10/713	Large-scale training	[22]
AssemblyNet	3D UNet	3x3x3x125<br>32x48x32x2 cubes	135	45/NA/19	125 UNets<br>Extra pre/post processing	[23]
SLANT	3D UNet	86x110x78x8 cubes<br>96x128x88x27 cubes	133	5111+45/NA/5+27+13	Extra preprocessing	[24]
ParcelCortex	3D UNet	96x96x48x2	62<br>130<br>96	2300/NA/41	Down-sampling<br>Multi-atlas hemisphere	[25]
FastSurfer	2.5D	256x256 patches	95	140/20/649		[26]

Table A.5. Runtime of FreeSurfer 7.1 version. We calculate the runtime of FreeSurfer using GARD cohort data including those not involved in training, and validating the DeepParcellation model. We could not clearly observe a linear relation between the runtime of FreeSurfer and the number of threads. Mean hours are denoted with a 95% confidence interval.

# threads	# samples	Mean hours	Min. hours	Max. hours
1	6821	13.22±0.07	5.48	38.35
16	15	3.56±0.18	3.20	4.55
24	15	9.4±0.14	9.04	9.89
100	5	8.43±3.49	6.99	13.44
168	2	11.14±37.29	8.20	14.07
256	3	4.98±7.09	3.17	8.27

Table A.6. Characteristics of training datasets.

Dataset	Partition	# Samples	# Subjects
ADNI	Train	808	764
ADNI	Val	113	96
ADNI	Test	110	96
ADNI	Total	1,031	956
GARD	Train	5,392	4,028
GARD	Val	909	503
GARD	Test	865	504
GARD	Total	7,166	5,035
Mindboggle101	Train	0	0
Mindboggle101	Val	0	0
Mindboggle101	Test	101	101
Mindboggle101	Total	101	101
OASIS	Train	1,263	1,263
OASIS	Val	158	158
OASIS	Test	158	158
OASIS	Total	1,579	1,579
SALD	Train	0	0
SALD	Val	0	0

SALD	Test	487	487
SALD	Total	487	487
TFU	Train	0	0
TFU	Val	0	0
TFU	Test	140	140
TFU	Total	140	140
ALL	Train	7,463	6,055
ALL	Val	1,180	757
ALL	Test	1,861	1,486
ALL	Total	10,504	8,298

Reproduction steps

Prerequisites

You should install CUDA-enabled GPU cards with at least 8GB GPU memory manufactured by nVidia, e.g., Titan XP.

Prepare T1-weighted MR images

1. Convert MR images to Neuroimaging Informatics Technology Initiative (NIfTI) format.
2. The parent directory name of a NIfTI file path will be used as **Subject Id** during prediction.
3. You can specify either **an input path** of the NIfTI file or **input direcotry** of many NIfTI files.

Install DeepParcellation

1. Install Anaconda
   - Download an Anaconda distribution: Link
2. Create a Conda environment

```
conda create -n deepparc python=3.8 -y
```

3. Install DeepParcellation (CPU mode)

```
conda activate deepparc
pip install deepparcellation
```

4. Install DeepParcellation (GPU mode)

```
conda activate deepparc
pip install deepparcellation
conda install cudnn=7.6.5 -c anaconda -y
conda install cudatoolkit=10.1.243 -c conda-forge -y
pip uninstall tensorflow -y
pip install tensorflow-gpu==2.2.0
pip uninstall keras -y
conda install keras-gpu=2.4.3 -c anaconda -y
```

5. Install DeepParcellation (Mac with M1 Chip)

```
conda activate deepparc
export GRPC_PYTHON_BUILD_SYSTEM_OPENSSL=1
export GRPC_PYTHON_BUILD_SYSTEM_ZLIB=1
export CFLAGS="-I/opt/homebrew/opt/openssl/include"
export LDFLAGS="-L/opt/homebrew/opt/openssl/lib"
conda install -c apple tensorflow-deps -y
pip install tensorflow-macos
pip install deepparcellation
```

6. Run DeepParcellation

```
conda activate deepparc
deepparcellation -o=/tmp/test --i=./subject-0-0000/test.nii.gz
or
deepparcellation -o=/tmp/test --i=./
```

NOTE:

1. You must always **activate the conda enviroment** before running DeepParcellation if you opened a **new console**.
2. You can see the source code of the package in the author's github (https://github.com/abysslover/deepparcellation)

References

1. Choi, H. & Jin, K. H. Fast and robust segmentation of the striatum using deep convolutional neural networks. *Journal of Neuroscience Methods* **274**, 146–153 (2016).

2. Dey, R. & Hong, Y. CompNet: Complementary Segmentation Network for Brain MRI Extraction. *Lecture Notes in Computer Science (including subseries Lecture Notes in Artificial Intelligence and Lecture Notes in Bioinformatics)* **11072 LNCS**, 628–636 (2018).
3. Thyreau, B., Sato, K., Fukuda, H. & Taki, Y. Segmentation of the hippocampus by transferring algorithmic knowledge for large cohort processing. *Medical Image Analysis* **43**, 214–228 (2018).
4. Zhang, W. *et al.* Deep convolutional neural networks for multi-modality isointense infant brain image segmentation. *NeuroImage* **108**, 214–224 (2015).
5. Stollenga, M. F., Byeon, W., Liwicki, M. & Schmidhuber, J. Parallel Multi-Dimensional LSTM, With Application to Fast Biomedical Volumetric Image Segmentation. *Advances in Neural Information Processing Systems* **2015-Janua**, 2998–3006 (2015).
6. Chen, H., Dou, Q., Yu, L., Qin, J. & Heng, P. A. VoxResNet: Deep voxelwise residual networks for brain segmentation from 3D MR images. *NeuroImage* vol. 170 446–455 (2018).
7. Li, J., Yu, Z. L., Gu, Z., Liu, H. & Li, Y. MMAN: Multi-modality aggregation network for brain segmentation from MR images. *Neurocomputing* **358**, 10–19 (2019).

8. Shakeri, M. *et al.* Sub-cortical brain structure segmentation using F-CNN'S. in *Proceedings - International Symposium on Biomedical Imaging* vols 2016-June 269–272 (IEEE Computer Society, 2016).
9. Milletari, F. *et al.* Hough-CNN: Deep learning for segmentation of deep brain regions in MRI and ultrasound. *Computer Vision and Image Understanding* **164**, 92–102 (2017).
10. Fedorov, A. *et al.* End-to-end learning of brain tissue segmentation from imperfect labeling. in *Proceedings of the International Joint Conference on Neural Networks* vols 2017-May 3785–3792 (Institute of Electrical and Electronics Engineers Inc., 2017).
11. Roy, A. G. *et al.* Error corrective boosting for learning fully convolutional networks with limited data. in *Lecture Notes in Computer Science (including subseries Lecture Notes in Artificial Intelligence and Lecture Notes in Bioinformatics)* vol. 10435 LNCS 231–239 (Springer Verlag, 2017).
12. Wachinger, C., Reuter, M. & Klein, T. DeepNAT: Deep convolutional neural network for segmenting neuroanatomy. *NeuroImage* **170**, 434–445 (2018).
13. Dolz, J., Desrosiers, C. & Ben Ayed, I. 3D fully convolutional networks for subcortical segmentation in MRI: A large-scale study. *NeuroImage* **170**, 456–470 (2018).

14. Guha Roy, A., Conjeti, S., Navab, N. & Wachinger, C. QuickNAT: A fully convolutional network for quick and accurate segmentation of neuroanatomy. *NeuroImage* **186**, 713–727 (2019).

15. Jog, A., Hoopes, A., Greve, D. N., Van Leemput, K. & Fischl, B. PSACNN: Pulse sequence adaptive fast whole brain segmentation. *NeuroImage* **199**, 553–569 (2019).

16. Sun, L. *et al.* A 3D Spatially Weighted Network for Segmentation of Brain Tissue from MRI. *IEEE Transactions on Medical Imaging* **39**, 898–909 (2020).

17. Lee, N., Laine, A. F. & Klein, A. Towards a deep learning approach to brain parcellation. in *Proceedings - International Symposium on Biomedical Imaging* 321–324 (2011). doi:10.1109/ISBI.2011.5872414.

18. De Brébisson, A. & Montana, G. Deep neural networks for anatomical brain segmentation. in *IEEE Computer Society Conference on Computer Vision and Pattern Recognition Workshops* vols 2015-Octob 20–28 (IEEE Computer Society, 2015).

19. Moeskops, P. *et al.* Automatic Segmentation of MR Brain Images with a Convolutional Neural Network. *IEEE Transactions on Medical Imaging* **35**, 1252–1261 (2016).

20. Li, W. *et al.* On the Compactness, Efficiency, and Representation of 3D Convolutional Networks: Brain Parcellation as a Pretext Task. *Lecture Notes in Computer Science*

(including subseries Lecture Notes in Artificial Intelligence and Lecture Notes in Bioinformatics) **10265 LNCS**, 348–360 (2017).

21. Mehta, R., Majumdar, A. & Sivaswamy, J. BrainSegNet: a convolutional neural network architecture for automated segmentation of human brain structures. *Journal of Medical Imaging* **4**, 024003 (2017).
22. Rajchl, M., Pawlowski, N., Rueckert, D., Matthews, P. M. & Glocker, B. NeuroNet: Fast and Robust Reproduction of Multiple Brain Image Segmentation Pipelines. *International conference on Medical Imaging with Deep Learning (MIDL) 2018* (2018).
23. Coupé, P. *et al.* AssemblyNet: A Novel Deep Decision-Making Process for Whole Brain MRI Segmentation. in *Lecture Notes in Computer Science (including subseries Lecture Notes in Artificial Intelligence and Lecture Notes in Bioinformatics)* vol. 11766 LNCS 466–474 (Springer, 2019).
24. Huo, Y. *et al.* 3D whole brain segmentation using spatially localized atlas network tiles. *NeuroImage* **194**, 105–119 (2019).
25. Thyreau, B. & Taki, Y. Learning a cortical parcellation of the brain robust to the MRI segmentation with convolutional neural networks. *Medical Image Analysis* **61**, 101639 (2020).

26. Henschel, L. *et al.* FastSurfer - A fast and accurate deep learning based neuroimaging pipeline. *NeuroImage* **219**, 117012 (2020).
